# Mechanistic model of phase-transitioning therapeutics injected into poroelastic tissue for improved targeting of superficial tumors

**DOI:** 10.21203/rs.3.rs-7900123/v1

**Published:** 2025-12-05

**Authors:** Daniel R. Adrianzen Alvarez, Eva S. Landeta Orozco, Nimmi Ramanujam, Jenna L. Mueller, David F. Katz

**Affiliations:** 1 Department of Biomedical Engineering, Duke University, Durham, NC, USA.; 2 Department of Bioengineering, University of Maryland, College Park, MD, USA.; 3 Duke Global Health Institute, Duke University, Durham, NC, USA; 4 Department of Pharmacology and Cancer Biology, Duke University, Durham, NC, USA; 5 Department of OB-GYN & Reproductive Science, University of Maryland School of Medicine, Baltimore, MD, USA.; 6 Marlene and Stewart Greenebaum Cancer Center, University of Maryland School of Medicine, Baltimore, MD, USA.; 7 Department of Obstetrics and Gynecology, Division of Gynecologic Oncology, Duke University Medical Center, Durham, North Carolina, USA.

**Keywords:** Intratumoral injection, phase transitioning therapeutic, drug delivery, injection delivery parameters, mechanistic modeling

## Abstract

The development of new drugs and drug delivery systems relies heavily on careful acquisition and interpretation of large amounts of experimental data, to identify and select promising candidates for therapeutic and prophylactic use. Predictive mathematical modeling can expedite this process by capturing the complex interplay of physical, chemical and biological factors that influence drug delivery. However, traditional compartmental models of pharmacokinetics and pharmacodynamics typically rely on oversimplified approximations of drug transport mechanisms and may fail to accurately represent the key deterministic processes that drive drug mass transport - particularly in complex delivery scenarios where the drug targets are close to the sites of drug administration. Here, we present a deterministic mathematical framework that addresses a challenging drug delivery modality: the injection of a fluid drug vehicle that undergoes phase separation upon entering poroelastic tissue. This phase change improves localized retention of the loaded drug in a finite volume near the injection site. Our model is directly relevant to in situ-gelling injections of chemotherapeutic agents into superficial tumors - a strategy gaining attention in the development of improved cancer therapeutics. Our approach uniquely incorporates both diffusion and convection, accounts for tissue poroelasticity, and uses Cahn-Hilliard theory to describe the phase separation behavior of the injected material. Simulations across a broad parameter space indicate that drug retention is enhanced in softer tissues and with high-rate, low-volume injections. This computational framework is currently being used to guide the design of improved therapeutic strategies for ethanol-based ablation, when co-injected with ethyl cellulose as a phase-transitioning agent for superficial tumors.

## Introduction

As the global incidence of solid tumor cancers continues to rise [1], there is growing demand for more effective and accessible treatments for unresectable tumors. In particular, localized treatment modalities, such as ablative therapies and intratumoral sustained-release formulations, have emerged as promising alternatives to systemic treatments, due to their ability to concentrate therapeutic agents at the tumor site and limit off-target effects [2–6]. However, these approaches remain difficult to optimize experimentally. Delivery outcomes depend on the complex interplay of host tissue mechanics, injection parameters, and the physiochemical properties of the active pharmaceutical agents (APIs). For example, tissue elasticity, interstitial fluid pressure, and drug diffusivity, influence the spatial distribution and retention of injected agents [7,8]. Further, injection parameters such as injectate properties, flow rate and volume, affect tissue deformation and subsequent API pharmacokinetics (PK) [9–13]. In addition, the molecular properties of APIs are central to their transport in tissue[7]. Given the complex, multivariate, non-linear interactions across these many causative factors, experimental development of intratumorally administered drugs must navigate a large, challenging parameter space.

Animal models used in preclinical development add further complications and limitations. While small animal models offer advantages such as cost-effectiveness, genetic homogeneity and ease of handling, their biological relevance and predictive power for human clinical outcomes remain fundamentally limited [14,15]. For example, differences in drug metabolism and elimination between humans and rodents can have substantial differences in PK and pharmacodynamic (PD) outcomes, complicating early-stage drug efficacy and toxicity assessments[15,16]. Large animal models have greater anatomical and often physiological relevance to humans, but their use in early drug development is constrained by stringent ethical factors, heterogeneity across small sample sizes, and high costs [14,17]. Consequently, new therapeutics are often developed and tested initially in suboptimal animal models with limited physiological relevance to humans[18].

Predictive mathematical modeling can help address these challenges. In silico models enable high-throughput exploration of complex parameter spaces, objective assessment of causative factors and governing mechanisms, and identification of salient variables affecting drug delivery outcomes [19]. For example, physiologically-based pharmacokinetic (PBPK) and PKPD models have significantly improved animal-to-human translation, and have contributed to rational design of both drugs and delivery mechanisms[20–22]. However, these approaches are generally tailored for parenteral or oral systemic delivery routes, where drugs are intended to reach distant organs via systemic circulation. In contrast, the topical, localized delivery problem addressed here - where the therapeutic target is located near the injection site, presents distinct biophysical and physicochemical challenges that demand a different modeling approach.

Deterministic modeling, adhering to basic principles of mass transport, offers a robust alternative. Unlike empirical compartmental PK models -which assume spatial homogeneity and rely heavily on fitted parameters that often lack physical interpretability –deterministic models are grounded in first-principle physical laws. They solve coupled partial differential equations that explicitly account for spatial and temporal variation in drug transport. This mechanistic formulation reduces dependence on dataset-specific calibration, minimizing the risk of over-fitting and improving generalizability across tissues, formulations, and species [30]. Furthermore, deterministic models can incorporate complex behaviors of drug vehicles, such as shear-thinning properties and phase separation, which significantly influence transport kinetics and local drug retention. The latter phenomenon is of central importance in the present study.

The modeling presented here is directly motivated by our ongoing work to develop a novel localized ablation strategy for superficial tumors using an ethyl cellulose - ethanol (EC-ethanol) solution and co-injected chemotherapeutic agents [12,23]. Upon injection into tissue, the dilute EC solution undergoes a phase transition in response to water, forming a cross-linked gel that enhances local drug retention at the tumor site and minimizes leakage to the surrounding tissues. Ethanol itself has ablative properties [23], and co-delivery with chemotherapeutic agents offers a synergistic therapeutic effect [12]. We have evaluated this ablative methodology across multiple species, identifying key parameters that govern injectate localization and ablation efficacy [23–25]. A predictive computational model capable of simulating this process would significantly accelerate its development, by guiding experimental design and parameter optimization.

Previous models have examined fluid injections in tissue, including some which account for poroelastic tissue deformation [26–28]. However, to our knowledge, none have incorporated drug vehicles which undergo phase transitions during the injection process. Modeling such vehicles, e.g. *in situ*-gelling hydrogels, presents a significant challenge, as their spatiotemporally-dependent phase separation dynamics alter drug transport kinetics and enhance drug retention at the injection site [23]. A comprehensive model of intratumoral, hydrogel-based drug delivery must capture the interplay of tissue deformation, diffusion-convection of the active pharmaceutical agent (API), and dynamic phase separation of the vehicle.

In this study, we present the first deterministic model of tissue injection that couples biphasic poroelastic tissue deformation, convection-diffusion mass transport, and vehicle phase separation dynamics. Tissue is modeled as a biphasic system with a poroelastic solid matrix and an interstitial fluid phase [29–31]. Drug transport is governed by coupled diffusion-convection processes [26–28], while vehicle phase separation is modeled using Cahn-Hillard theory, which describes the spontaneous formation of distinct phases driven by chemical potential gradients [32–34]. This integrated model provides a mechanistic framework for quantifying how tissue properties, injection parameters, and drug formulation physicochemical characteristics influence spatiotemporal drug distribution and retention.

Our approach represents a significant advancement in modeling localized drug delivery with in-situ gelling vehicles, such as hydrogels, which are gaining increasing attention in oncology [12, 34–36]. By capturing key features —including tissue deformation, pressure-driven flow phase-separation, and spatiotemporal evolution of injectate properties — our model can be applied and extended broadly to inform and support rational design and optimization of intratumoral therapies.

## Materials and Methods

### Model Overview of an Intratumorally Injected Phase-Transitioning Therapeutic

Intratumoral injection creates elevated hydrostatic pressure at the needle tip, deforming the surrounding poro-viscoelastic tissue and forming a transient cavity filled with the injected therapeutic material. The injectate consists of the API and its delivery vehicle. The dimensions of the cavity are governed by a combination of factors, including the injectate’s viscosity, the mechanical and transport properties of the tissue (such as porosity, elasticity and permeability), and user-controlled injection parameters (e.g. flow rate, volume and needle gauge).

In the case of phase transitioning therapeutics, the delivery vehicle includes a sustained-release engendering (SR) agent that responds to environmental cues —commonly temperature, pH, light or hydration. In this study, we focus on SR agents that gel upon contact with the aqueous environment of biological tissue. As the injection progresses, both tissue deformation and mass transport are dynamically modulated by the evolving phase transitioning behavior of the SR agent.

**Figure 1:**
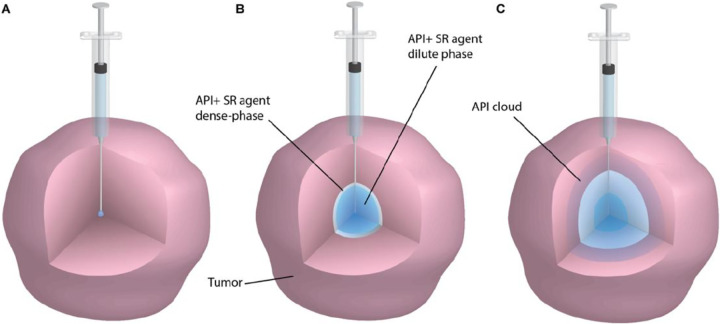
Illustration of the injection process over time. (A) At t=0, the needle is inserted into the tissue, the initial cavity radius is equal to the inner radius of the needle. (B) At an intermediate time during the injection, the cavity radius has grown, and the SR has phase-separated. A shell along the boundary of the cavity and a liquid core full of the API have formed. (C) The API has moved through the dense phase of the SR agent into the surrounding tissue, via convection and diffusion. At the end of the injection process, an API cloud has formed that continues to evolve in space and time.

At the onset of injection, a small spherical cavity forms at the needle tip, with an initial radius equal to the needle’s inner radius (Fig 1A). As fluid continues to flow through the needle, this cavity expands outwards, while gelation begins at the interface between injectate and the surrounding tissue (Fig 1B). The resulting structure consists of a liquid core – containing the API and dilute phase (ungelled) SR – surrounded by a dense gel shell. Through convection and diffusion, the API then migrates outward: first through the dense-phase gel layer, and subsequently into the tissue itself, forming an API diffusion cloud (Fig 1C).

**Figure 2:**
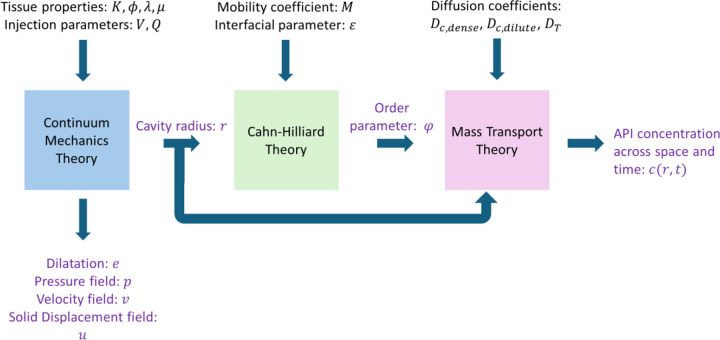
Computational model schematic. The three mathematical theories integrated in the computational model. Arrows indicate the parameter’s direction in and throughout the model. The inputs are show in black, and outputs in purple. See Table 1 for symbol definitions.

To model this process, we developed an integrated mathematical framework that couples three key mechanisms: (1) soft tissue deformation; (2) phase separation and gelation of the SR agent; and mass transport of the API via diffusion and convection (Fig 2). These interacting phenomena are governed by a system of coupled partial and ordinary differential equations, which we solved numerically to compute the spatiotemporal evolution of drug concentration distribution.

### Model Assumptions

To construct a tractable, physiologically relevant model, we made key assumptions across mechanical, transport and phase-separation domains. We assumed that the tissue behaves as an isotropic, linearly elastic material, consistent with previous poroelastic modeling studies [26]. This approximation is most appropriate for relatively small deformations, and allows us to treat the tissue’s hydraulic conductivity, K as a constant. Importantly, we also assumed that the injection pressure remains below the tissue’s critical pressure, which prevents fracturing and allows us to model the system as a one-dimensional, spherically symmetric biphasic problem [26]. Cases involving tissue rupture and leakage — when cavity pressure exceeds the critical threshold — are thus outside the scope of this model.

The injection is divided in two sequential phases: (1) an active fluid injection phase, during which a fixed volume V is delivered at a constant flow rate Q; and (2) a passive relaxation phase initiated when the injection ends at time t = V/Q. During the active phase, tissue deformation is pressure-driven and governed by Q, the hydraulic conductivity K, and the tissue’s elastic properties — specifically, Lamé’s parameters μ and λ. Here, μ represents the shear modulus (resistance to shape deformation) and λ characterizes resistance to volumetric compression; together these parameters are related to the Young’s modulus and Poisson ratio of the tissue. We assumed that the injected SR agent precipitates instantaneously into its dense phase upon contacting tissue, forming a gel at the cavity boundary. Convective transport of the API itself depends on tissue solid displacement rates via the fluid velocity $v_{f}$.

The tissue is modeled as semi-permeable: it allows diffusion and convection of the API but is impermeable to the SR agent due to the high molecular weight of polymeric SR molecules. The diffusion coefficient of the API is constant in tissue. However, in the cavity, the phase separation of the SR agent alters the local diffusivity: the denser the gel (quantified by the order parameter φ), the lower the API’s mobility. This is implemented as a linear reduction in diffusion coefficient with increasing SR density. After the injection ends, we assume that the velocity drops to negligible levels instantaneously, and that the cavity radius remains fixed due to the mechanical support of the gelled SR agent.

### Continuum Mechanics: Biphasic Poroelastic Tissue Deformation

This model follows the mathematical framework originally developed by Mow et al. and expanded upon by Netti et al., where tissue is modeled as a biphasic system made up of solid and fluid phases. The governing equations, Cauchy stress tensors, and constitutive equations are given by finite deformation biphasic theory [26], [35].

Mass Balance:

(1) $$\nabla \cdot \left(\Phi v_{f}+(1-\Phi )\frac{\partial u}{\partial t}\right)=0$$

Momentum Balance:

(2) $$\nabla \cdot \sigma^{f}+\nabla \cdot \sigma^{s}=0$$

(3) $$\Phi \left[v_{f}-\frac{\partial u}{\partial t}\right]=-K\nabla p$$

The fluid phase stress tensor consists of a hydrostatic term (equation 4), while the solid phase also incorporates and elastic term described by the Lamé parameters μ and λ, the deformation tensor ϵ, and the solid dilatation e (equation 5). e is the trace of ϵ (equation 6), where the deformation tensor ϵ is given by equation 7. All constitutive equations are listed below:

(4) $$\sigma^{f}=-\Phi \text{pI}$$

(5) $$\sigma^{s}=-(1-\Phi )\text{pI}+2\mu \epsilon +\lambda \text{eI}$$

(6) $$e=\text{tr}(\epsilon )=\nabla \cdot u=\frac{\partial u_{r}}{\partial r}+\frac{2u_{r}}{r}$$

(7) $$\epsilon =\frac{1}{2}\left[\nabla u+(\nabla u{)}^{T}\right]$$

As boundary conditions, we impose negligible dilatation at the outer margin of the domain, r=R (equation 8), and constant velocity at the initial radius, r = r_0_, given by the flow rate and the initial surface area of the cavity across which flow occurs (equation 9). Finally, the partial solid stresses on the surface of the cavity, $\tau_{\text{rr}}$, at r = r_0_, are set to zero (equation 10). As an initial condition, dilatation is set to zero everywhere (equation 11).


(8) e(r=R,t)=0

(9) $$v_{f}\left(r=r_{0},t\right)=\frac{Q}{4\pi r_{0}^{2}}$$

(10) $$\tau_{\text{rr}}\left(r=r_{0},t\right)=2\mu \frac{\partial u_{r}\left(r=r_{0},t\right)}{\partial r}+\lambda e\left(r=r_{0},t\right)=0$$

(11) e(r,t=0)=0

The system of equations was solved to obtain the solid displacement field u, the dilatation of the solid matrix e, the pressure field P, and the fluid velocity $v_{f}$ (see below).

### Cahn-Hillard Theory: Phase Separation of the SR Agent

The phase separation behavior of the SR agent was modeled using Cahn Hilliard theory, a continuum, phase-field approach used to describe phase separation and coarsening in binary systems [36]. The order parameter, φ(r,t), represents the local composition of the SR agent, and evolves over space and time according to the Cahn-Hilliard equation. This order parameter φ is defined within the range φ∈[-1,1], where φ(r,t)=-1 corresponds to the SR agent in its pure liquid (dilute) phase and φ(r,t)=1 corresponds to the fully gelled (dense) phase. This continuous variable allows computation of phase-dependent properties—such as the local diffusion coefficient of the API- across spatial and temporal domains.

The dynamics of phase separation are governed by Equation 12, where the rate of phase separation is modulated by the mobility coefficient M. Precipitation of the SR agent is driven by the chemical potential $\mu_{\varphi }$, which reflects the change in free energy of the system and determines the thermodynamic driving force for phase separation (equation 13). The interfacial energy parameter ε penalizes sharp gradients and stabilizes the interface between phases, controlling how diffuse or sharp the interface is. The total free energy of the system is described by a double-well potential function F(φ), which is typical of binary mixtures (equation 14).


(12) $$\frac{\partial \varphi }{\partial t}(r,t)=\nabla \cdot \left[M(\varphi (r,t))\nabla \mu_{\varphi }(r,t)\right]$$

(13) $$\mu_{\varphi }(r,t)=F^{\prime }(\varphi (r,t))-\varepsilon^{2}\Delta \varphi (r,t)$$

(14) $$F(\varphi (r,t))=0.25{\left(\varphi (r,t{)}^{2}-1\right)}^{2}$$

As a boundary condition, we set φ=1 at the cavity-tissue interface to represent instantaneous SR phase separation (equation 15). Inside the cavity, we set φ=-1 everywhere to represent a cavity that is initially filled with dilute-phase SR (equation 16).


(15) $$\varphi \left(r=r_{c},t\right)=1$$

(16) $$\varphi \left(r<r_{c},t=0\right)=-1$$

### Mass transport: Mass Transport via Convection-Diffusion

The transport of the API was modeled using the common convection-diffusion equation (equation 17); where the fluid velocity $v_{f}$ is presented in the continuum mechanics section above. This modeling applies during both the active injection and post-injection relaxation phases, though in the latter we assume $v_{f}$ drops to 0.

The diffusion coefficient of the API within the cavity was modeled as a function of the order parameter φ(r,t), which encodes the local phase state of the SR agent. In the two limiting cases, φ=1 (fully gelled/dense phase) and φ=-1 (dilute/liquid phase), the diffusion coefficients were defined as $D_{c,\text{dense}}$ and $D_{c,\text{dilute}}$ respectively. For intermediate values of φ in the cavity, the effective diffusion coefficient D(φ) was taken as a linear function of it (equation 18). In the surrounding tissue, where SR does not penetrate, the diffusion coefficient was assumed to be constant (equation 19).


(17) $$\frac{\partial c}{\partial t}(r,t)=\nabla \cdot [D(\varphi (r,t))\nabla c(r,t)]-\nabla \cdot \left(v_{f}c\right)$$

(18) $$D(\varphi (r,t))=\frac{1}{2}\left(D_{c,\text{dense}}-D_{c,\text{dilute}}\right)(\varphi +1)+D_{c,\text{dilute}},r\leq r_{c}$$

(19) $$D(\varphi (r,t))=D_{t},r>r_{c}$$

At the boundary between the cavity and the surrounding tissue ($r=r_{c}$), we imposed a concentration partition condition to account for potential variations in API solubility across the interface (equations 20–21).

For the initial condition, the API concentration was set to a constant loaded value within the needle, and set to zero elsewhere (equations 22–23).


(20) $$c\left(r={r_{c}}^{-},t\right)=P\cdot c\left({r_{c}}^{+},t\right)$$

(21) $$D_{c}\left(\varphi \left(r={r_{c}}^{-},t\right)\right)\frac{\partial c}{\partial r}\left(r={r_{c}}^{-},t\right)=D_{T}\frac{\partial c}{\partial r}\left(r={r_{c}}^{+},t\right)$$

(22) $$c\left(r>r_{0},t=0\right)=0$$

(23) $$c\left(r\leq r_{0},t\right)=c_{0},0\leq t\leq \frac{V}{Q}$$

### Parameter Value Selection

The injection process involves multiple coupled physical phenomena and parameter dependencies (Table 1). To model a generalizable API-SR injection scenario, we guided our parameter selection using prior experimental studies of ethanol (as the API) and ethyl cellulose (as the SR agent) injections[23], [25], with additional parametric sweeps to explore variations in formulation, tissue properties and injection dynamics (Table 3). While the model is adaptable to specific drugs and formulations, our primary aim was to establish a framework applicable across a range of conditions; therefore, certain parameters were estimated or idealized based on physiochemical analogs or scaling arguments.

The mobility coefficient M and interfacial parameter ε govern the kinetics of phase separation and interface formation during gelation. These were estimated empirically by fitting our simulated order parameter evolution (given all other baseline parameter values) to match the time to complete gelation observed in prior ethanol-ethyl cellulose injections into agarose phantoms and murine breast cancer models [11], [24],47]. In those studies, we observed that full cavity gelation typically occurred within 5–15 minutes, depending on EC concentration. While theoretical approaches exist to relate M and ε to mixture thermodynamics and interfacial tension, we opted for empirical fitting to reproduce physiologically relevant timeframes. Further discussion on generalizing these values for other API-SR mixtures is provided in the Discussion.

Anatomically, we assumed a spherically symmetric tumor with a diameter of a = 1cm, consistent with large murine breast cancer models [47]. We imposed needle placement at its center. To capture both intratumoral and peritumoral transport, we extended the total simulation domain to a radius of 2cm, encompassing both the cavity, tumor, and surrounding healthy tissue.

We modeled diffusive transport using distinct diffusion coefficients in the dilute (pre-gelation) and dense (gelled) phases of the SR matrix, as well as for the surrounding tissue. Ethanol in water has a known diffusion coefficient of $\sim 10^{-5}{cm}^{2}{\text{s}}^{-1}$ [37]. To account for the increased viscosity of the SR formulation (e.g. a 10-fold increase in viscosity from water to 3% ethyl cellulose in ethanol[23]), we applied the Stokes-Einstein relation to estimate that the diffusion coefficient of ethanol in the dilute phase was one order of magnitude lower, $D_{c,\text{dilute}}=10^{-6}cm^{2}s^{-1}$.

We previously observed that the addition of 6% ethyl cellulose to ethanol reduces the effective diffusion coefficient in tissue by approximately half [25]. This measured effective diffusion coefficient $D_{\text{eff}}≅\frac{D_{T}}{2}$ reflects both the slowed release of ethanol from the phase-separated ethyl-cellulose-filled cavity and its transport through surrounding tissue. Assuming two radial resistances in series—first through the dense cavity material, and then through tissue—we defined the effective radial resistance $\omega_{\text{eff}}$ as:

(24) $$\omega_{\text{eff}}=\frac{a}{D_{\text{eff}}}=\int_{0}^{r_{c}}\frac{dr}{D_{c,\text{dense}}}+\int_{r_{c}}^{a}\frac{dr}{D_{T}}$$

Using this relationship, and the assumption that $r_{c}≅0.1a$, we estimated $D_{c,\text{dense}}\approx 0.09\;D_{T}$. To account for the possibility of denser or more tightly crosslinked SR networks in experimental settings (e.g. higher concentrations of ethyl cellulose), and to test a suitable maximal range of values, we selected $D_{c,\text{dense}}=0.02{\;D}_{T}$ as a baseline for subsequent simulations.

Our model supports a discontinuous partitioning interface between the cavity and tissue characterized by partition coefficient P. For simplicity, we set P=1 implying that the API is equally soluble in both compartments. This is reasonable for ethanol, given its low molecular weight, high aqueous solubility and absence of known binding interactions in soft tissue. For APIs with preferential solubility or tissue binding, this coefficient can be readily adjusted.

We began our analysis by selectively varying mechanical tissue properties to assess their influences on solid deformation. Tissue permeability K was swept from 3.8e^−11^ m^2^/kPa to 7.6e^−10^ m^2^/kPa, and Lame’s second parameter μ was increased up to one order of magnitude above reference values to represent stiffer tumor matrices. Injection volume V and the flow rate Q were varied to explore injection parameter effects on cavity size and API retention. Finally, to test how phase separation dynamics and matrix resistance impact transport, we swept M by two orders of magnitude and $D_{c,\text{dense}}$ by one order of magnitude above and below their reference values

### Numerical Solution Strategy

To obtain numerical solutions, we first addressed the continuum mechanics equations governing tissue deformation by applying Laplace transforms, enabling derivation of closed-form expressions in the Laplace domain. These expressions were then numerically inverted using an inverse Laplace transform solver implemented in MATLAB (MATLABR2023a, The MathWorks Inc., Natick, MA, U.S.A.). For the remaining coupled partial differential equations, including those governing phase separation and API mass transport, the spatial domain was discretized into 1,000 points. The equations were then recast into finite difference form. A backward difference scheme was applied in the domain $0\leq r\leq r_{c}$, corresponding to the interior of the injection cavity, while a central difference scheme was used in the region $r_{c}\leq r\leq R$, where R denotes the outer boundary of the simulation domain. This hybrid approach was chosen to enhance numerical stability, particularly near the moving cavity boundary. The full system was subsequently integrated using MATLAB’s stiff ODE solver ode15s, which is well-suited for systems exhibiting rapid changes in solution gradients.

## Results

We evaluated how four categories of determinants influenced API distribution and retention: (1) host tissue properties, including stiffness (Lamé parameters) and hydraulic conductivity; (2) injection parameters, including flow rate, volume, and needle radius; (3) phase separation behavior of the SR agent, governed by the mobility coefficient and interfacial parameter; and (4) API transport properties, including diffusion coefficients in both the dense and dilute SR phases and in tissue. Determinants (3) and (4) are especially dependent on interactions between the injected material and the host tissue environment.

For all simulations, we used representative parameter values based on our EC–ethanol system for localized tumor ablation. Sensitivity analyses were performed on each group of determinants while holding others at their reference values. Model outputs included spatiotemporal maps of API concentration and the normalized mass fraction of API retained within the cavity and tumor tissue (hereafter referred to as “API retention”).

### API Retention in the Cavity and Tumor was Increased in Soft and Impermeable Tissue (Figures 3 and 4)

To assess how tissue properties influence API retention, a sensitivity analysis was performed by varying two parameters: hydraulic conductivity K and Lamé’s second parameter μ, which respectively characterize tissue permeability and stiffness. One parameter was varied at a time while holding others constant, and the results were compared.

**Figure 3:**
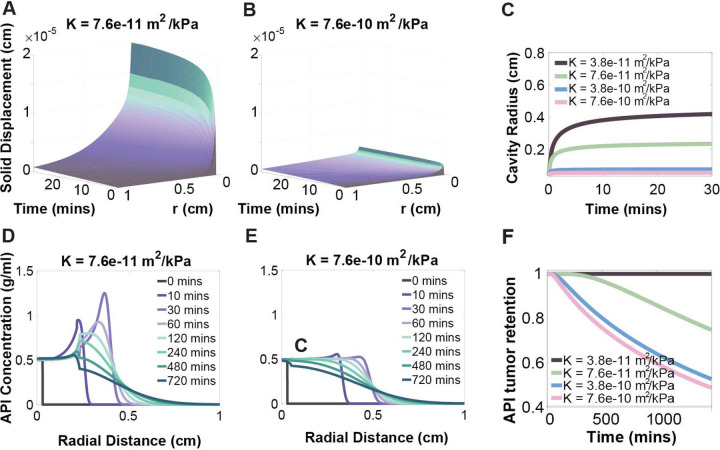
Hydraulic conductivity effects on solid displacement and cavity radius. (A and B) The solid deformation fields for tissues at different hydraulic conductivities within the tumor region: (A) K= 7.6×10^−11^ m^2^/kPa; and (B) K= 7.6×10^−10^ m^2^/kPa). (C) Cavity radius over time for different hydraulic conductivities within the tumor region (range: K= 7.6×10^−10^– 3.8×10^−11^ m^2^/kPa). Cavity radius was calculated as $r_{c}=r_{0}+u\left(r=r_{0}\right)$, where $u\left(r=r_{0}\right)$ is the magnitude of the radial solid displacement field at the initial cavity boundary r_o_. (D and E) Representative API concentration plots vs. radial distance at different time points for different hydraulic conductivities with the tumor region(D) K= 7.6×10^−11^ m^2^/kPa; (E) K= 7.6×10^−10^ m^2^/kPa. (F) Normalized fraction of API mass in cavity and tumor over time for tissues with different hydraulic conductivities (range: K= 7.6×10^−10^– 3.8×10^−11^ m^2^/kPa).

Higher hydraulic conductivity values were associated with reduced tissue deformation and smaller cavity radii (Fig 3A–C), due to greater fluid permeability. This allowed dispersal of the injectate into surrounding tissue, thereby limiting deformation. API transport was also faster in tissues with higher hydraulic conductivity, resulting in lower API retention within both the cavity and the tumor tissue over time (Figures 3D–F).

**Figure 4:**
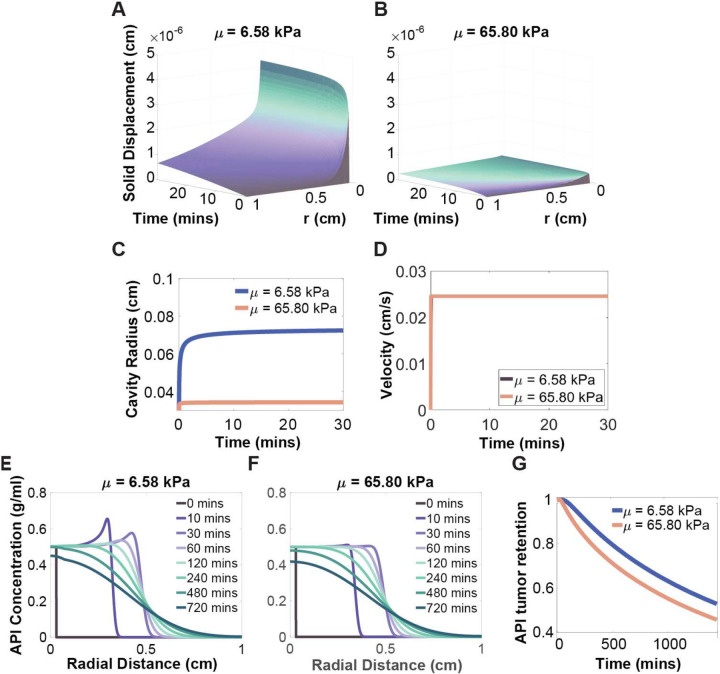
Tissue stiffness effects on solid displacement and cavity radius. (A and B) The solid deformation fields with the tumor region for tissues with different elasticities (varying Lamé’s second parameter: (A) μ=6.58kPa; (B) μ=65.8kPa). (C) Cavity radius over time for different tissue elasticities. (D) Velocity over time for different values of lame’s second parameter. (E and F) Representative API concentration plots vs. radial distance at different time points for different hydraulic conductivities within the tumor region: (E) μ=6.58kPa, F: μ=65.8kPa); (G) Normalized fraction of API mass in cavity and tumor over time for tissues with different elasticities (μ=6.58-65.80kPa).

Increasing the value of Lamé’s second parameter, which is proportional to the material’s Young’s modulus, reflects stiffer tissue, and similarly reduced tissue deformation and decreased cavity radius (Fig 4A–C). Although the velocity field ${\text{v}}_{\text{f}}$ in tissue was independent of μ (Fig 4D), the API distribution was sensitive to tissue stiffness (Fig 4E–F). Specifically, the model predicted reduced API retention in stiffer tissues with higher values of μ (Fig 4G). In smaller cavities there was a smaller effective transport distance, accelerating API permeation and ultimately decreasing retention in both the cavity and tumor. Overall, API retention was maximized in soft, impermeable tissue, with $K\leq 3.8\times 10^{-11}\;{\text{m}}^{2}/\text{kPa}$ and μ≤6.58kPa.

### Lower Injection Volumes at High Flow Rates Increased API Retention (Figures 5 and 6)

Next, a sensitivity analysis was conducted to evaluate how injection volume and flow rate affect API retention. Pressure and velocity was examined over time at the needle tip $\left(r=r_{0}\right)$, where magnitudes were highest.

**Figure 5:**
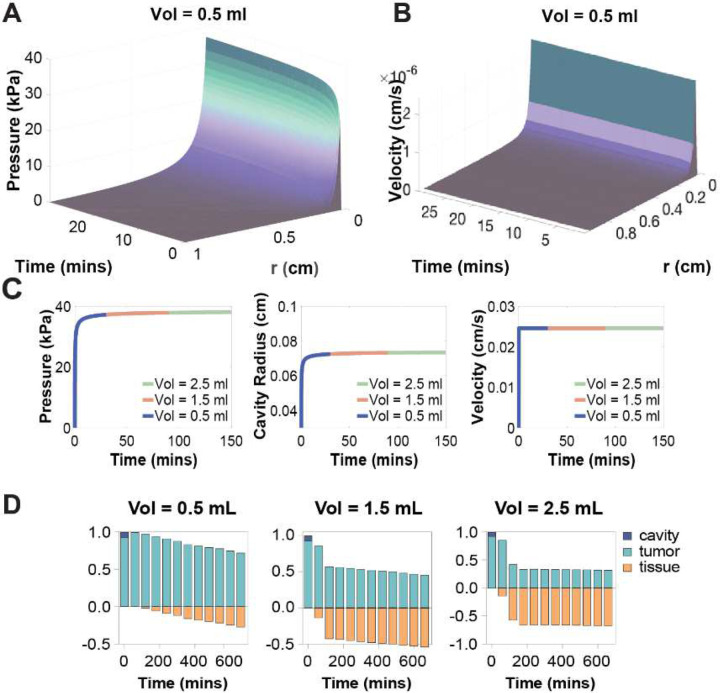
Effects of volume on pressure, velocity, cavity radius and API distribution. (A) Representative pressure field for an injection volume of 0.5mL. (B) Representative velocity field for an injection volume of 0.5mL. (C) Pressure, cavity radius, and velocity over time for different injection volumes (range 0.5–2.5ml). (D) Normalized API mass distribution in cavity, tumor tissue, and healthy tissue over time at different injection volumes (range 0.5–2.5mL).

In general, the model predicted that pressure and velocity increased over time and decreased with radial distance (Fig 5A and 5B). When the flow rate was held constant, increasing the injection volume had minimal impact on peak pressure, cavity radius or velocity (Fig 5C), but prolonged the injection duration. This extension increased the duration of convective transport, allowing more API to escape the cavity and resulting in decreased retention (Fig 5D).

**Figure 6:**
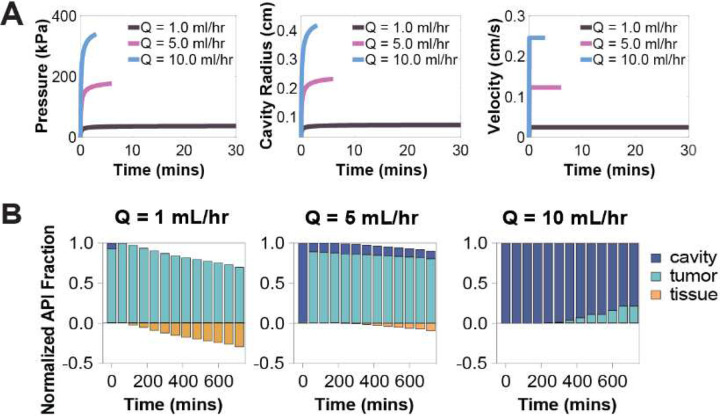
Effects of injection rate on pressure, velocity, cavity radius and API distribution. (A) Pressure, cavity radius, and fluid velocity over time for different injection rates (range 1–10mL/hr). (B) Normalized API mass distribution in cavity, tumor tissue, and healthy tissue over time at different injection rates (range 1–10mL/hr).

In contrast, increasing the injection flow rate (at constant volume) led to higher transient pressures, larger cavities, and elevated fluid velocities, but over a shorter injection duration (Fig 6A). This rapid injection phase resulted in improved API retention in both the cavity and tumor tissue (Fig 6B). An injection rate of 5mL/hr was found to produce the highest overall retention within the target tissue.

### SR Agents with Lower Mobility and Dense-Phase Diffusion Coefficients Improved API Retention in Tumor Tissue, but Had Limited Influence (Figures 7 and 8)

**Figure 7:**
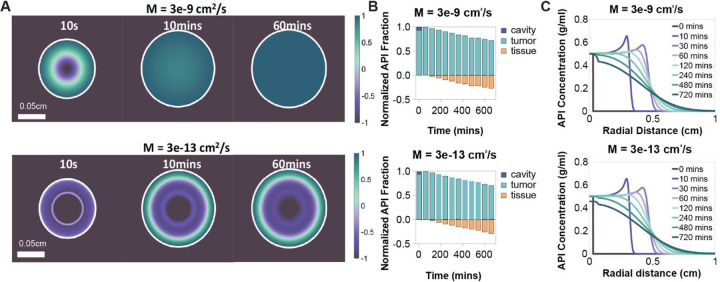
Effects of mobility coefficient on phase order and API concentration distribution. (A) Representative heatmaps of the Cahn-Hillard order parameter over the course of the injection process (t=10s, 10mins, 60mins) at different mobility coefficients. The white circle is the tumor-tissue boundary. 1 corresponds to the dense phase of the SR release agent and −1 to the dilute phase. (B) Normalized API distributions in the cavity, tumor tissue, and healthy tissue for different mobility coefficients (Top: M= 3e-9 cm^2^/s and Bottom: M= 3e-13 cm^2^/s). (C) Concentration of API vs. radial distance within the tumor region for different mobility coefficients (Top: M= 3e-9 cm^2^/s and Bottom: M= 3e-13 cm^2^/s) at different times points.

To evaluate how SR phase separation affects drug delivery, we varied the mobility coefficient M across five orders of magnitude, from 3×10^−9^ to 3×10^−13^ cm^2^/s. Significant differences in phase separation dynamics were observed over time. Increasing M accelerated SR gelation, resulting in a larger fraction of the injection depot transitioning to the dense phase earlier during injection (Fig 7A). However, this had negligible effect on API concentration profiles or normalized mass distribution (Fig 7B–C), likely because convection dominated transport, causing the API to exit the cavity at a faster rate than the rate of gelation.

**Figure 8:**
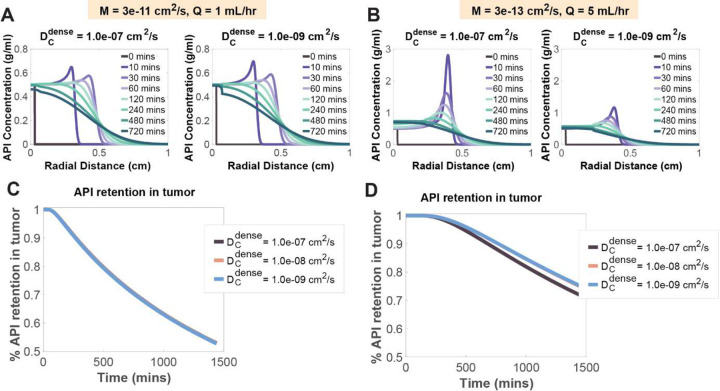
Effects of the diffusion coefficient in the dense phase on API tumor concentration distribution. (A) API concentration distribution vs. radial distance within the tumor region for different dense phase coefficients at different time points with a flow rate of 1 mL/hr and M = 3e-11 cm^2^/s (Left: ${\text{D}}_{\text{c},\text{dense}}=1\text{e}-7\;{\text{cm}}^{2}/\text{s}$ and Right: ${\text{D}}_{\text{c},\text{dense}}=1\text{e}-9\;{\text{cm}}^{2}/\text{s}$). (B) API concentration distribution vs. radial distance within the tumor region for different dense phase coefficients at different time points with a flow rate of 5 mL/hr and M = 3e-13 cm^2^/s (Left: ${\text{D}}_{\text{c},\text{dense}}=1\text{e}-7\;{\text{cm}}^{2}/\text{s}$ and Right: ${\text{D}}_{\text{C},\text{dense}}=1\text{e}-9\;{\text{cm}}^{2}/\text{s}$). (C) Normalized fraction of API mass in tumor at different dense phase coefficients over time for Q= 1 mL/hr and M = 3e-11 cm^2^/s. (D) Normalized fraction of API mass in tumor at different dense phase coefficients over time for Q= 5 mL/hr and M = 3e-13 cm^2^/s.

The dense-phase diffusion coefficient was also varied to assess its effect on API transport. During the injection phase, convection rapidly cleared a large proportion of the API from the cavity, leaving a limited amount for diffusion to act upon. As a result, the dense-phase diffusion coefficient had a minimal influence on API concentration, normalized mass distribution, or overall retention (Fig 8A,C). However, under conditions of elevated flow rate (>5 mL/hr) and low mobility coefficient (3×10^−13^ cm^2^/s), the effect of the dense-phase diffusion coefficient became more pronounced (Fig 8B–D). Higher flow rates produced larger cavities, increasing the transport distance between the cavity center and surrounding tumor tissue. In this scenario, the larger gel volume allowed the dense-phase diffusion coefficient to modulate transport more effectively, resulting in modest improvements in API retention.

## Discussion

The development of new therapeutic agents and their delivery vehicles remains heavily reliant on experimental methodologies, which are often time-consuming, resource-intensive, and limited in their ability to resolve mechanistic detail [38]. Computational models, particularly those predicting PK and PD, can improve this process by enabling systematic exploration of complex, multivariate parameter spaces and the underlying mechanisms governing drug transport and distribution [39].

Standard compartmental PK models are not designed to capture the heterogeneous concentration gradients and tissue-scale mechanics that govern localized intratumoral injections. To address this gap, we developed a deterministic model that integrates poroelastic deformation, convection–diffusion drug transport, and phase-separation dynamics of the delivery vehicle. This framework enables mechanistic evaluation of how injection parameters, tissue mechanics, and the behavior of phase-separating SR agents influence drug retention in the target volume. Key model outputs include spatiotemporal maps of API concentration, which were summarized as total API mass retained in the target tissue over time.

The model provided several key mechanistic insights with direct implications for intratumoral therapy design. First, API retention was highest in soft, low-permeability tissues. Stiffer tissues, characterized by higher Lamé parameters, resisted deformation more strongly during injection, resulting in smaller cavities and greater fluid displacements into surrounding tissue, thereby decreasing API retention. For example, increasing μ tenfold from 6.58kPa to 65.8kPa reduced API tumor retention by ~14% after 24 hours. This prediction is consistent with known mechanical barriers to drug transport in solid tumors, where unregulated cell proliferation and extracellular matrix remodeling (e.g. elevated collagen and hyaluronan) increase stiffness, reduce porosity, and impair interstitial transport [7, 40–41]. Similarly, lower hydraulic conductivity improved retention by limiting convective drug loss, consistent with experimental observations in dense tumor tissues [42].

Second, the model predicted that lower injection volumes delivered at higher flow rates improved local retention. Higher flow rates increased cavity expansion, confining the injectate closer to the delivery site, while smaller volumes shortened the injection phase (t=V/Q), limiting the time available for convective loss. For instance, reducing the injection volume from 2.5mL to 0.5mL at a constant flow rate increased tumor retention significantly, by ~2.5x after 12 hours. These findings are corroborated by experimental data showing that low volumes and higher flow rates improve injectate retention in agarose phantoms, *ex vivo* tissue, and various animal tumor models [43–45]. Together, they suggest that clinically, optimizing injection protocols towards smaller volumes and faster rates could substantially enhance localized drug exposure while minimizing leakage to surrounding tissues.

The model predicted that the phase-separation parameters of the SR agent -specifically the mobility coefficient and the dense-phase diffusivity – had minimal impact on API retention. This can be attributed to three interacting factors. First, the boundary condition imposed on the Cahn Hilliard equation enforced instantaneous phase separation at the cavity interface, forming a thin, dense-phase shell. Due to the cavity’s rapid expansion, the rate of shell thickening during cavity growth outpaced the mobility-limited phase separation kinetics, thereby diminishing the influence of the mobility coefficient. Second, API transport during injection was dominated by convection, which displaced most of the API before the evolving gel structure could substantially influence retention. Third, the SR agent was assumed to remain confined within the cavity (even in its dilute phase) due to its high molecular weight, preventing it from permeating into tissue. As a result, gelation effects were spatially constrained. While this assumption is appropriate for many polymeric formulations, cases where SR partially permeates tissue may require alternative boundary conditions, such as flux boundary conditions. These findings underscore the importance of aligning gelation kinetics with injection dynamics: if the gel does not form quickly enough to immobilize the API, convection will dominate transport and negate the potential gains in retention that may occur with denser gels.

One notable discrepancy between our model and experimental findings involves the role of injection volume. Some experimental studies report that larger injection volumes increase local pressures and cavity radii in hydrogel-based systems [12], [43], whereas our model predicted minimal changes in cavity size or pressure beyond a threshold volume. This mismatch likely reflects five simplifying assumptions. First, tissue was modeled using linear elasticity, which does not account for the strain-stiffening behavior of biological soft tissues. This likely led to an underestimation of pressure buildup at larger deformations. Second, the SR agent was assumed to remain fully contained in the cavity, eliminating potential coupling between gelation, tissue deformation and transport properties outside of the cavity. Third, the model treated cavity pressure as spatially uniform and omitted momentum balances that would account for mechanical stresses arising due to gelation. This allowed the cavity to expand passively, without developing internal stresses during phase-separation. Fourth, hydraulic conductivity was assumed constant, despite its known dependence on tissue strain and microstructural remodeling. Real tissues often exhibit strain-dependent permeability, where deformation alters pore structure and modulates resistance to flow, resulting also in pressure buildup. Lastly, the model did not incorporate tissue fracture, which can occur when local pressure exceeds a critical threshold. Such fractures create low-resistance escape paths that abruptly relieve pressure and alter fluid flow and drug distribution. While our design goal is to avoid fracture during injection, understanding its onset is important for accurate modeling of pressure-volume relationships and drug retention. Including fracture mechanics in future models may clarify the conditions under which gel-based SR systems enhance retention by plugging leakage pathways or by increasing mechanical resistance to fluid escape. Future work should consider incorporating these mechanisms to improve alignment with experimental findings.

Additional limitations of the model include the assumptions of spherical symmetry, tissue homogeneity, and linear biphasic elasticity. Real tumors are mechanically heterogenous and anisotropic. These features can lead to asymmetric injectate distributions and variable resistances to cavity expansion. In addition, while poroelasticity is appropriate for the longer injection timescales considered here, viscoelastic effects may become important for shorter or faster injections. Further, phase separation was treated as decoupled from mechanical resistance. This may hold for soft gels such as those formed by EC-ethanol systems, but stiffer gels could interact with deformation and alter pressure dynamics. Despite these simplifications, the present model captures essential features absent from prior formulations and provides actionable guidance for optimized localized injections.

This study presents the first computational model that integrates phase-separating injectable formulations with poroelastic tissue mechanics and API mass transport. By incorporating Cahn-Hilliard theory into a biphasic continuum framework, the model provides a mechanistic, tunable platform for exploring how injection parameters, tissue properties and gelation kinetics govern API retention across a wide range of applications and injection characteristics. The results highlight the benefits of using lower injection volumes and higher flow rates, and underscore the importance of matching gelation kinetics to injection dynamics. By incorporating increasingly realistic features, such as anisotropic tissue structure, viscoelastic deformation and tissue fracture, future iterations of this model will better support the rational design of localized drug delivery systems for clinical use.

## Figures and Tables

**Table 1.** Key Parameters and Outputs of the Model

Key Inputs	Outputs
Tissue properties	Continuum mechanics theory
Hydraulic conductivity, K Lame's first and second parameters, λ and μ Porosity, ϕ	Cavity radius r Dilatation e Pressure field p Velocity field v
Injection characteristics	Cahn-Hilliard theory
Volume, V Flow rate, Q Needle internal radius, $r_{0}$	Order parameter maps φ
	Mass transport theory
	Spatiotemporal API concentration c Normalized API mass fraction in the cavity, target tissue and surrounding healthy tissue
SR agent phase-change properties	
Mobility coefficient, M Interfacial parameter, ε	
Transport properties	
Diffusion coefficient of API in tissue, $D_{T}$ Diffusion coefficient of API in SR agent dense phase, $D_{c,\text{dense}}$ Diffusion coefficient of API in SR agent dilute phase, $D_{c,\text{dilute}}$	

**Table 2.** Summary of Model Assumptions

Domain	Assumption
Continuum Mechanics	Tissue is isotropic and linearly elastic Injection pressure remains below tissue’s critical pressure Injectate volume V is delivered at a fixed rate Q Velocity drops instantaneously to negligible levels at t=V/Q Tissue deformation is a function of Q, K, μ and λ Velocity field $v_{f}$ is driven by the rate of solid displacement Cavity radius remains constant during relaxation
Cahn-Hilliard Phase Separation	SR agent instantaneously gels at the cavity-tissue boundary Cavity initially contains SR entirely in its dilute phase
Mass Transport	Tissue is semi-permeable to the API, impermeable to SR agent. API transport is governed by convection and diffusion Diffusivity is constant in tissue, but varies inside cavity API diffusion decreases linearly with increasing SR density (i.e. the order parameter φ).

**Table 3.** Reference Values of Parameters used in the Injection Model

Description	Symbol	Value	Source
Hydraulic conductivity in tissue	K	$3.8\text{e-}10{\text{m}}^{2}/\text{kPa}$	[26]
Tissue porosity	ϕ	0.2	[26]
Lamé’s first parameter	λ	13.16kPa	[26]
Lamé’s second parameter	μ	6.58kPa	[26]
Flow rate	Q	1mL/hr	[11], [23]
Injection volume	V	0.5mL	[24]
Needle internal radius	$r_{0}$	0.03cm	[26]
Mobility coefficient	M	$3\text{e-}11{\text{cm}}^{2}/\text{s}$	Derived from preliminary sample injections
Interfacial parameter	ε	0.2	Derived from preliminary sample injections
Diffusion coefficient in tissue	$D_{T}$	$5\text{e-}7{\text{cm}}^{2}/\text{s}$	[25]
Diffusion coefficient in dense phase of gelling agent	$D_{c,\text{dense}}$	$1\text{e-}8{\text{cm}}^{2}/\text{s}$	Expected to be significantly lower than diffusion coefficient of ethanol in water
Diffusion coefficient in dilute phase of gelling agent	$D_{c,\text{dilute}}$	$1\text{e-}6{\text{cm}}^{2}/\text{s}$	Expected to be lower than diffusion coefficient of ethanol in water
Partition coefficient at cavity boundary	P	1	Derived from ethanol’s solubility and binding affinity
Initial radius of tumor	a	0.5cm	[25]
Total dimension of simulation domain	R	2cm	N/A

## Data Availability

The computational model developed is openly available at https://github.com/danieladrianzenSoft/InjectionModelWithGelation. All data presented in this manuscript was directly outputted by this model.
